# Risk factors and prediction model of urosepsis in patients with diabetes after percutaneous nephrolithotomy

**DOI:** 10.1186/s12894-021-00799-3

**Published:** 2021-04-28

**Authors:** Jun Liu, Qingya Yang, Jiayi Lan, Yang Hong, Xiaobo Huang, Bo Yang

**Affiliations:** 1 Urology and Lithotripsy Center, Peking University People’s Hospital, 133 Fuchengmen Inner Street, Xicheng District, Beijing, 100034 People’s Republic of China; 2 Peking University Applied Lithotripsy Institute, Peking University, Beijing, 100034 People’s Republic of China; 3 Department of Urology, Qilu Hospital (Qingdao), Cheeloo College of Medicine, Shandong University, Qingdao, 266035 People’s Republic of China

**Keywords:** PCNL, Diabetes, Urosepsis, Risk factors, Nomogram

## Abstract

**Objective:**

To analyze the risk factors of patients with diabetes mellitus (DM) and urosepsis after percutaneous nephrolithotomy (PCNL) for upper urinary tract stones and to develop a nomogram to predict postoperative urosepsis according to the risk factors.

**Methods:**

The data of patients with type 2 diabetes who underwent one-stage PCNL due to upper urinary tract stones were retrospectively analyzed. The risk factors of patients with postoperative urosepsis were evaluated by univariate and multivariate logistic regression analysis, and the nomogram prediction model was developed according to the regression coefficient.

**Results:**

One-stage PCNL was successfully completed in 241 patients with DM, and urosepsis occurred in 41 (17.0%) patients after PCNL. Based on multivariate logistic regression analysis, the independent risk factors associated with postoperative urosepsis included preoperative leukocyte elevation (OR = 3.973, P = 0.005), positive urine nitrite (OR = 3.697, P = 0.010), and positive urine culture (OR = 3.562, P = 0.002). According to the results of the logistic regression analysis model, staghorn stones (OR = 2.049, P < 0.1) and complete intraoperative stone clearance (OR = 0.431, P < 0.1), were used to develop the nomogram. Internal validation of the nomogram showed that the concordance index (C-index) was 0.725. Additionally, the Hosmer–Lemeshow test was performed, P = 0.938 > 0.05.

**Conclusion:**

Preoperative leukocyte elevation, positive urine nitrite, and positive urine culture are independent risk factors for urosepsis after one-stage PCNL for patients with DM with upper urinary tract stones. The nomogram, which is based on independent risk factors that combine stone morphology and intraoperative stone clearance, can help predict the risk of postoperative urosepsis.

## Introduction

After decades of development, percutaneous nephrolithotomy (PCNL) has become one of the main surgical methods for upper urinary tract stones, especially those with kidney stones or upper ureteral stones larger than 2 cm [1]. However, it is important to consider the potential postoperative complications caused by PCNL [2, 3].

Urosepsis is a serious postoperative complication of PCNL. If it is not properly treated in a timely manner, it can cause septic shock and can be lethal [4]. Clinical studies have shown that diabetes mellitus was independently associated with increased risk of infectious complications after PCNL(OR = 14.6, P = 0.001) [5]. Therefore, when implementing PCNL in patients with DM, early detection of patients with potential urosepsis risk is extremely important. This study retrospectively analyzed clinical data of patients with DM submitted for primary PCNL treatment for upper urinary tract stones at the Urology and Lithotripsy Center of Peking University People's Hospital, as well as the independent risk factors for urosepsis after PCNL. Subsequently, a prediction model for postoperative urosepsis was developed for early detection of urosepsis in patients with DM when performing PCNL, as well as prevention and early intervention.

## Materials and methods

### Patients

We retrospectively analyzed the data of patients who underwent one-stage PCNL for upper urinary tract stones and were preoperatively diagnosed with type 2 diabetes mellitus(T2DM) at the Urology and Lithotripsy Center of Peking University People's Hospital between June 2012 and December 2019. Diagnosis of T2DM was defined by 2-h PG(two-hour 75-g postload plasma glucose level) ≥ 11.1 mmol/L on OGTT(oral glucose tolerance test) or FPG(fasting plasma glucose) ≥ 7.0 mmol/L according to ADA(American Diabetes Association) criteria, or a previous diagnosis of T2DM. Exclusion criteria included (1) patients who underwent bilateral endoscopic lithotripsy for one-stage surgery; (2) patients with severe systemic diseases, namely, American Society of Anesthesiologists (ASA) class IV-VI status; (3) solitary kidney and patients undergoing nephrectomy; and (4) patients with missing data. Clinical data of 241 consecutive patients were collected.

### Clinical data

Preoperative preparation included blood cell count, serum biochemical parameters, renal ultrasound, abdominal X-ray examination, and urinary CT examination if necessary. After perineal cleaning, midstream urine was collected for routine urine test and urine culture. Positive urine white blood cells (WBC) were defined as WBC 10 per high power field (× 400). For patients with positive WBC or nitrite in urine, antibiotics were given immediately for 3–7 days and adjusted according to the results of the urine culture and drug sensitivity test during treatment. Regardless of whether the preoperative urine culture was positive or negative, all patients received a single dose of antibiotic prophylaxis 30 min before PCNL and continued to use it 48 h after surgery if the urine culture was positive. Body temperature, blood pressure, respiration, heart rate, white blood cells, and serum creatinine were closely monitored after PCNL.

Upper urinary tract stones occurring in the renal pelvis and extending to at least two calyces are classified as staghorn stones. All patients were followed up with systemic inflammatory response syndrome (SIRS) criteria after surgery (white blood cell count < 4000 × 10^9^/L or > 12,000 × 10^9^/L; fever > 38 °C or < 36 °C; heart rate > 90 beats/min; respiratory rate > 20 breaths/min), and blood culture was tested if necessary [6]. SIRS was diagnosed in patients who met two or more criteria. Urosepsis diagnostic criteria included both urinary tract infections and SIRS, while other site infections were excluded [4, 7].

### Surgical methods

All patients received spinal anesthesia. A retrograde 5-Fr ureteral catheter (BARD, USA) was inserted into the affected side, with a 22-Fr cystoscope in the lithotomy position and a 16-Fr ureteral catheter was inserted for bladder drainage. After ureteral catheter insertion, the patient was placed in a prone position and underwent renal puncture under the guidance of 3.5 MHz ultrasound at the 11th intercostal space, or the 12th subcostal space, using a 18-gauge puncture needle. After observing urine flow through the needle, a J-shaped guide wire was placed into the collection system through a puncture needle, and the nephrostomy tract was dilated with a nested metal dilator until a 24 Fr Amplatz sheath was inserted. A 20.8Fr rigid nephroscope (Richard Wolf, Knittlingen, Germany) combined with ultrasonic and pneumatic lithotripsy (Swiss lithotripter, EMS, Nyon, Switzerland) was used for stone fragmentation in all cases. Intraoperative ultrasonography, or X-ray fluoroscopy, was used to check whether the kidney stones were completely cleared, and stone clearance was defined in cases in which fragments of stones were less than 4 mm. A 6-Fr double J catheter and a 14-Fr nephrostomy tube (BD Medical System, Franklin Lake, New Jersey, USA) were placed at the end of each surgery. Additionally, vital signs were monitored 24 h after PCNL and fasting peripheral blood routines were performed the next morning to help diagnose SIRS. The duration of the surgery was obtained from the patient's surgical anesthesia record sheet, in which the start of the procedure consisted of the ultrasound-guided renal puncture and the end of the surgery consisted of the placement of the indwelling nephrostomy tube.

### Statistical methods

The data were statistically analyzed using SPSS20.0 software. Continuous variables were tested for normality, and those conforming to normal distribution were expressed by mean ± standard deviation and analyzed by independent sample *t*-test; those not conforming to normal distribution were expressed by median (minimum, maximum), together with ordered categorical variables, and the comparison between groups was expressed by Mann–Whitney U-test analysis. The chi-square test analysis was used for comparison between groups of disordered categorical variables; univariate and multivariate logistic regression analysis was used for risk factor analysis. A P < 0.05 was considered statistically significant.

Based on the results of the logistic regression analysis model, and combined with clinically significant variables, the nomogram prediction model was constructed using the RMS software package of R software (R 3.6.2 Institute of Statistics and Mathematics, Vienna, Austria). The bootstrap method was used to sample 1000 times repeatedly, and the nomogram model was verified internally. Model accuracy was evaluated by calculating the consistency index (C-index), drawing the calibration curve, and by the Hosmer–Lemeshow goodness-of-fit test [8].

## Results

### Clinical data and surgical outcome

One-stage PCNL was successfully completed in 241 patients with DM, including 135 males and 106 females, with ages ranging from 28 to 81 years, with mean age of 57 years, mean stone size of 2.96 ± 2.18 cm, and a median operative time of 70 (20–175) min. Urosepsis occurred in 41 (17.0%) patients after PCNL. All cases of urosepsis occurred within 24 h postoperatively, and all cases improved after anti-infection treatment. The patients were classified in one of two groups: the urosepsis group and the non-urosepsis group, based on postoperative occurrence of urosepsis. The comparison of clinical parameters between the two groups is shown in Table 1. Compared with the non-urosepsis group, patients in the urosepsis group had a higher preoperative white blood cell count (× 10^9^/L) (8.8 ± 2.9 vs. 7.1 ± 2.3, P < 0.001) and longer hospital stay (13.7 ± 5.5 vs. 11.2 ± 5.2, P = 0.006). Also, we observed an increased incidence of urosepsis in female patients versus male patients (P < 0.05), and an increased incidence of urosepsis in patients with staghorn stones and residual stones (P < 0.05). Notably, there were no significant differences between the two groups regarding age, BMI index, blood glucose level, duration of surgery, and number of channels.

**Table 1:** Comparison of clinical data between patients with and without postoperative urosepsis

Items	Non-urosepsis	Urosepsis	P value
Gender, n(%)			
Male	118	17	0.039
Female	82	24	
Age/years	57.3 ± 9.7	55.4 ± 10.9	0.272
BMI (kg/m^2^)	26.2 ± 4.0	26.1 ± 4.2	0.814
Hypertension			
Yes	133	30	0.407
No	67	11	
Staghorn stone			
Yes	44	16	0.022
No	156	25	
Preoperative urine WBC			
Positive	173	40	0.045
Negative	27	1	
Preoperative urine nitrite			
Positive	27	15	0.0004
Negative	173	26	
Preoperative urine culture			
Positive	74	28	0.0002
Negative	126	13	
Preoperative blood WBC (109/L)	7.1 ± 2.3	8.8 ± 2.9	0.0001
Preoperative hemoglobin (g/L)	134.4 ± 18.5	132.7 ± 20.1	0.608
Preoperative serum creatine (μmol/L)	84.5 ± 39.6	92.4 ± 55.8	0.285
Preoperative blood glucose (mmol/L)	6.9 ± 2.1	7.2 ± 2.1	0.352
ASA			
I–II	187	35	0.079
III	13	6	
Operative time/min	60 (20–145)	75 (20–175)	0.349
Tract			
Single	174	31	0.063
Multiple	26	10	
Stone clearance			
Yes	178	30	0.007
No	22	11	
Hospital stay (days)	11.2 ± 5.2	13.7 ± 5.5	0.006

*BMI* body mass index, *WBC* white blood cell, *ASA* American Society of Anesthesiologists

### Logistic regression analysis of clinical features and urosepsis

In univariate analysis, we analyzed potential predictors based on three aspects, including patient, stone and surgery (Table 2). The results showed that the factors significantly correlated with postoperative urosepsis in univariate analysis included female (OR = 2.032, P = 0.042), preoperative WBC elevation (OR = 2.750, P = 0.019), preoperative positive urine culture (OR = 3.667, P < 0.001), positive urinary nitrite (OR = 3.697, P < 0.001), staghorn stones (OR = 2.269, P = 0.024), and intraoperative stone clearance (OR = 0.337, P = 0.009). These factors were included in the multivariate logistic regression analysis. The results showed that there were three variables that could be used as independent risk factors of postoperative urosepsis: preoperative WBC elevation (OR = 3.973, P = 0.005), positive urinary nitrite (OR = 3.697, P = 0.010), and positive urine culture (OR = 3.562, P = 0.002).

**Table 2:** Univariate and multivariate logistic regression analysis of clinical data and urosepsis

Variables	Univariate analysis			Multivariate analysis		
	OR	95% CI	P value	OR	95% CI	P value
Gender						
Male	Ref			Ref		
Female	2.032	1.027 to 4.019	0.042	0.681	0.271 to 1.710	0.413
Age						
> 60	Ref					
≤ 60	0.981	0.948 to 1.015	0.271			
BMI						
> 26	Ref					
≤ 26	0.990	0.909 to 1.077	0.813			
Preoperative blood glucose (mmol/L)						
< 7.0	Ref					
≥ 7.0	1.076	0.923 to 1.254	0.352			
Preoperative urine WBC						
Negative	Ref					
Positive	6.243	0.824 to 47.3134	0.076			
Preoperative urine nitrite						
Negative	Ref			Ref		
Positive	3.697	1.739 to 7.856	0.0007	3.697	1.360 to 10.050	0.010
Preoperative urine culture	Ref			Ref		
Negative						
Positive	3.667	1.789 to 7.517	0.0004	3.562	1.584 to 8.011	0.002
Preoperative WBC (≥ 10X109/L)						
No	Ref			Ref		
Yes	2.750	1.182 to 6.395	0.019	3.973	1.519 to 10.394	0.005
Staghorn stone						
No	Ref			Ref		
Yes	2.269	1.114 to 4.621	0.024	2.049	0.891 to 4.712	0.092
Stone clearance						
No	Ref			Ref		
Yes	0.337	0.148 to 0.766	0.009	0.431	0.162 to 1.142	0.091

### Nomogram for predicting the probability of postoperative urosepsis following PCNL

**Fig. 1:**
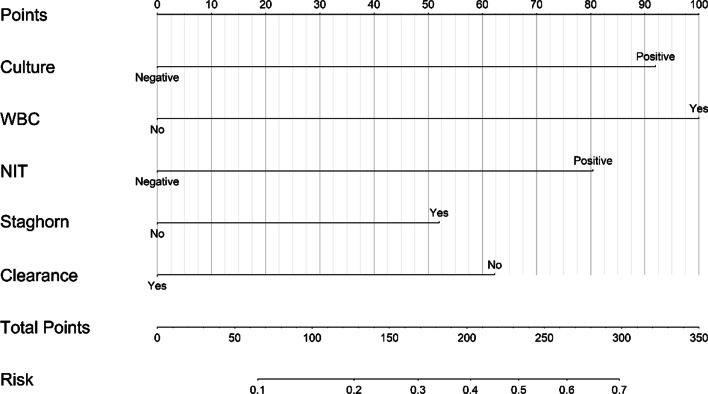
Nomogram for predicting the probability of postoperative urosepsis following PCNL in patients with DM

**Fig. 2:**
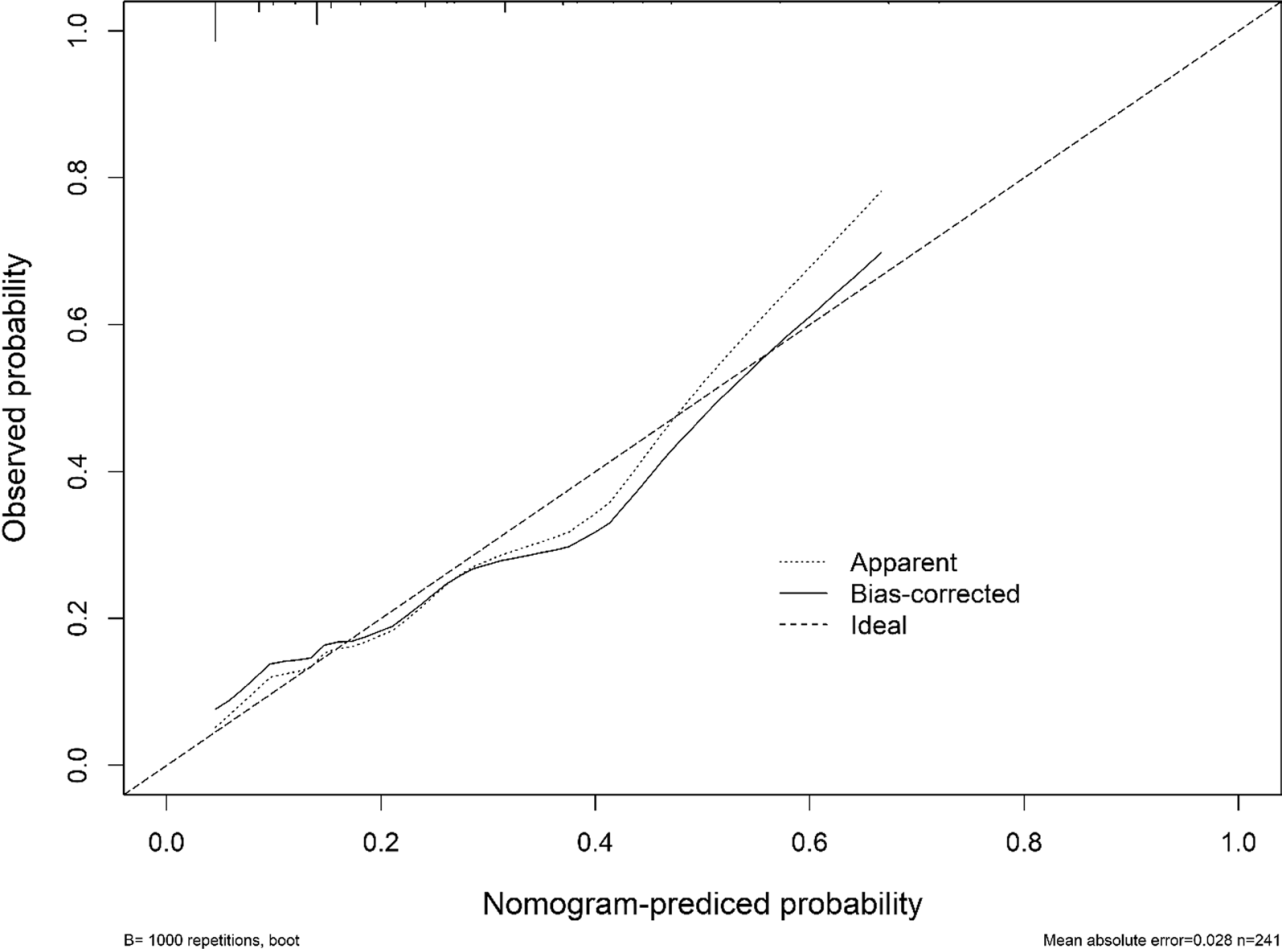
Calibration curve of the prediction model for postoperative urosepsis following PCNL in patients with DM

Based on multivariate logistic analysis, we included two parameters related to calculi, staghorn calculi (OR = 2.049, P < 0.1) and complete intraoperative stone clearance (OR = 0.431, P < 0.1), in the development of the nomogram (Fig. 1). Additionally, we performed the bootstrap method repeated sampling 1000 times and determined the internal validation of the nomogram model, with consistency index (C-index) of 0.725, and by drawing the Calibration curve. Calibration of the nomogram was close to the standard curve (Fig. 2), and the Hosmer–Lemeshow goodness-of-fit test was performed (P = 0.938 > 0.05), which indicated that the nomogram model could accurately predict the risk of urosepsis in patients with DM after PCNL surgery, and had good consistency with the actual risk.

NOMOGRAM USE INSTRUCTION: Use the corresponding points on each index axis on the nomogram to make a vertical line on the scoring scale to get the score of the index. After calculating the sum of the scores of each index, correspond downward to determine the risk of urosepsis in this patient after PCNL.

Culture: Preoperative urine culture; WBC positive: Preoperative blood WBC ≥ 10X10^9^/L; NIT: Preoperative urine nitrite; Staghorn: staghorn stone; Clearance: stone clearance.

## Discussion

The main objective of this retrospective study was to construct, and internally validate, a nomogram to predict the incidence of urosepsis after PCNL in patients with DM. To our knowledge, this is the first clinical retrospective study of a population of patients with DM undergoing PCNL, in which the associated risk factors can be obtained in the first postoperative period, leading to an earlier assessment of the risk of postoperative urosepsis for early intervention and treatment.

In recent years, with the development of minimally invasive technology, the safety and effectiveness of PCNL has improved; however, postoperative infection is still one of the major complications. Studies have shown that the incidence of infectious complications after PCNL surgery is 2.8–32.1% [9]. If postoperative infection is not detected in time and there is no active anti-infective treatment, septic shock can result. Indeed, in different regions and countries, mortality rates for severe sepsis and septic shock vary among medical centers, with reported results ranging from 22 to 76% based on available epidemiological data [10]. Urosepsis refers to sepsis due to urinary tract and/or infections of the male reproductive system [4, 7]. In recent years, research on risk factors of urosepsis caused by urinary calculi has increased significantly. However, most studies have focused on risk factors for urosepsis after endoscopic lithotripsy, such as procalcitonin levels, stone culture, and postoperative WBC levels [11, 12]. Kumar et al. demonstrated the importance of time as a prognostic factor: initiating empirical antibiotic therapy within an hour of diagnosis of sepsis was associated with 80% survival. For each hour of delayed antibiotic use, the survival rate decreased by an average of 7.6% (79.9% for 1–2 h, 42.0% for 5–6 h, and 25.4% for 9–12 h) [13]. Therefore, early identification and treatment of urosepsis have important clinical significance, which is also the aim of this study.

In our study, all patients were diabetic, with an overall incidence of urosepsis of 17.0%. Compared with 7.6–14.3% reported in the general population [4, 14], the incidence is relatively high, and it is currently believed that the reason for the increased incidence of postoperative infection caused by diabetes may be related to the following mechanisms. First, diabetes-related microvascular disease reduces oxygen and nutrient delivery to peripheral tissues [15]. Also, hyperglycemic status impairs the function of leukocytes and monocytes [16]. Additionally, peripheral neuropathy alters neuropeptide-mediated inflammatory response [17]. Therefore, for patients with DM receiving PCNL, it is important to identify the preoperative and intraoperative risk factors independently related to urosepsis after surgery in order to identify and implement the appropriate treatment as early as possible. Unfortunately, we did not find a statistical difference in preoperative blood glucose levels between the two groups. This result may be due to the fact that all patients with DM had their preoperative blood glucose level adjusted over a prolonged period of time before surgery, with surgery only being performed when the blood glucose level reached an acceptable range.

In this study, positive preoperative urine culture results were an independent risk factor for predicting urosepsis after PCNL in patients with DM (OR = 3.562, P = 0.002). However, current studies have confirmed that the sensitivity of preoperative midstream urine culture results for predicting postoperative SIRS is only 50% compared with pelvic urine culture and stone culture, and that it cannot predict the occurrence of postoperative SIRS [18]. However, both pelvic urine and stone culture require specimens to be obtained during surgery, and there is an increased wait time for results after surgery; therefore, effective intervention in cases of postoperative infection cannot be made in a timely manner, which limits the clinical application value of pelvic urine and stone cultures. Additional studies have shown that the significance of preoperative midstream urine culture cannot be ignored. Indeed, Gutierrez studied 865 patients undergoing PCNL and found that positive preoperative midstream urine culture (OR = 2.12, P < 0.05) was an independent risk factor for fever after PCNL [19]. Similarly, a multivariate analysis of 496 patients receiving ureteroscopic laser lithotripsy by Uchida et al. showed that obstructive pyelonephritis, positive preoperative bladder urine culture results, and female gender were associated with postoperative SIRS [20]. Our results are in line with results of previous studies that have demonstrated that positive preoperative midstream urine culture results are significantly associated with increased risk of urosepsis after surgery. Additionally, this result can be obtained preoperatively, which is extremely important for predicting the risk and for prevention of infection after PCNL.

In this study, multivariate analysis found that patients with preoperative WBC ≥ 10 × 10^9^/L had a 3.973-fold higher risk of urosepsis than patients with normal preoperative WBC (P = 0.005). Liang et al. conducted a retrospective analysis of 287 patients who underwent stone surgery and found that preoperative WBC (WBC > 10 * 10^9/L^) (P = 0.027), type of surgery (P = 0.009), intraoperative hypotension (P < 0.001), and emergency surgery (P < 0.001) were associated with intraoperative and postoperative urosepsis in calculous pyonephrosis patients.^4^ In addition, the same study also found that urine nitrite (NIT) positive patients had a 3.697-fold higher postoperative risk of urosepsis than NIT (–) patients (P = 0.010); therefore, urine nitrite status may help predict urinary tract infection and the need for subsequent treatment. Fan et al. conducted a multivariate logistic analysis of 156 patients undergoing minimal PCNL surgery and showed that preoperative positive NIT status (OR = 10.570, P = 0.025), stone size (OR = 11.512, P = 0.009), and postoperative leukopenia (OR = 0.009, P < 0.001) were independently associated with uroseptic shock [21]. These results are consistent with the results of this study, which provide a good reference for our early detection of urosepsis after PCNL.

In our study, staghorn stones and stone clearance were not independent risk factors for urosepsis after PCNL in multivariate regression analysis. Nevertheless, staghorn stones have always been difficult to treat in PCNL surgery. Also, the morphology of staghorn stones is complex, involving multiple calyces. It may take longer stone removal by lithotripsy during surgery, and it also increases the possibility of multiple tracts. Rivera analyzed several infectious factors after PCNL surgery and found that in multivariate analysis, the presence of staghorn stones alone was independently associated with an increased risk of postoperative infectious complications (OR = 3.14; P = 0.02) [22]. Similar studies in people with DM undergoing PCNL also reached the same conclusion. For example, Wei et al. showed by logistic regression analysis that diabetic complications and complete comorbidity of staghorn stones were independent risk factors for postoperative infectious complications [23]. Residual stones may contain bacteria and endotoxins and increase the risk of infectious complications after PCNL surgery [24]. The occurrence of intraoperative residual stones may be caused by various reasons, such as staghorn stones, renal structural abnormalities, calyceal neck stenosis, intraoperative bleeding, etc. The pursuit of full clearance may also increase the operation time and irrigating pressure, resulting in increased complications of infection. Therefore, while controlling the operation time and irrigating pressure, the stones should be completely removed as far as possible. To judge whether the stone is completely removed, we can use intraoperative ultrasonography and X-ray fluoroscopy to determine the need for interventions of anti-urosepsis after PCNL.

Based on the results of the above multivariate analysis, we included five risk factors associated with the occurrence of urosepsis after PCNL, including preoperative urine culture (P = 0.002), preoperative WBC elevation (P = 0.005), preoperative NIT (P = 0.010), staghorn stones (P = 0.092), and stone clearance (P = 0.091).These five factors may be interconnected; therefore, we constructed a nomogram according to the relative risk of each factor, which is convenient for rapid clinical reference and application. After internal verification, the C-index of this nomogram is 0.725, which has good conformity. The aforementioned five risk factors can be obtained in the first postoperative period; therefore, the risk of urosepsis in high-risk patients needs to be fully considered when making surgical plans. The application of this tool not only contributes to clinical decision-making and timely intervention for patients with potentially severe infections but also provides a referable digital basis for surgical prognosis and facilitates preoperative communication with patients regarding surgical options and possible risks, which is extremely important for perioperative management of the diabetic population. This study is also the first study to investigate the occurrence of urosepsis after PCNL in patients with DM.

This study has some limitations. First, this is a retrospective study and selection bias cannot be avoided. However, we carefully selected the inclusion criteria and collected sufficient clinical samples to enable patients in both the experimental and control groups to acquire an accurate representation of disease occurrence. Second, the data from the prediction model was obtained from a single center. Although we used patient samples from different periods, evidence from other centers is needed to validate the model. Therefore, we intend to carry out a multicenter study and provide sufficient clinical data to further evaluate and externally validate our prediction model. Despite these limitations, to our knowledge, this study presents the most critical assessment to date of urosepsis secondary to PCNL surgery in patients with DM and, for the first time, provides a nomogram that can be used in the first postoperative period.

## Conclusion

Preoperative WBC elevation, positive urine culture, and urine NIT( +) were independent risk factors for urosepsis in patients with DM after one-stage PCNL. We constructed a nomogram to predict urosepsis postoperatively, which can provide help predict the risk of urosepsis after PCNL in patients with DM and help individualized treatment of upper urinary tract stones.

## Data Availability

The datasets used and analysed during the current study are available from the corresponding author on reasonable request.

## Abbreviations

DM: Diabetes mellitus
PCNL: Percutaneous nephrolithotomy
T2DM: Type 2 diabetes mellitus
2-h PG: Two-hour 75-g postload plasma glucose level
OGTT: Oral glucose tolerance test
FPG: Fasting plasma glucose
ADA: American Diabetes Association
CT: Computed tomography
WBC: White blood cells
SIRS: Systemic inflammatory response syndrome
NIT: Urine nitrite
